# The diagnostic accuracy of contrast echocardiography in patients with suspected cardiac masses: A preliminary multicenter, cross-sectional study

**DOI:** 10.3389/fcvm.2022.1011560

**Published:** 2022-09-16

**Authors:** Ying Li, Weidong Ren, Xin Wang, Yangjie Xiao, Yueqin Feng, Pengli Shi, Lijuan Sun, Xiao Wang, Huan Yang, Guang Song

**Affiliations:** ^1^Department of Ultrasound, Shengjing Hospital of China Medical University, Shenyang, China; ^2^Department of Ultrasound, The First Hospital of China Medical University, Shenyang, China; ^3^Department of Ultrasound, The Fourth Affiliated Hospital of China Medical University, Shenyang, China; ^4^Department of Ultrasound, The First Hospital of Qinhuangdao, Qinhuangdao, China; ^5^Department of Ultrasound, Anshan Central Hospital, Anshan, China; ^6^Department of Ultrasound, Yingkou Central Hospital, Yingkou, China

**Keywords:** cardiac mass, heart neoplasms, echocardiography, ultrasound enhancing agents, sensitivity, specificity

## Abstract

**Background:**

To evaluate the diagnostic accuracy of contrast echocardiography (CE) in patients with suspected cardiac masses.

**Methods:**

A multicenter, prospective study involving 108 consecutive patients with suspected cardiac masses based on transthoracic echocardiography performed between November 2019 and December 2020 was carried out. CE examinations were performed in all patients. The echocardiographic diagnosis was established according to the qualitative (echogenicity, boundary, morphology of the base, mass perfusion, pericardial effusion, and motility) and quantitative (area of the masses and peak intensity ratio of the masses and adjacent myocardium A1/A2) evaluations.

**Results:**

Final confirmed diagnoses were as follows: no cardiac mass (*n* = 3), pseudomass (*n* = 3), thrombus (*n* = 36), benign tumor (*n* = 30), and malignant tumor (*n* = 36). ROC analysis revealed the optimal A1/A2 with cutoff value of 0.295 for a cardiac tumor from a thrombus, with AUC, sensitivity, specificity, PPV, and NPV of 0.958 (95% confidence interval (CI): 0.899–0.988), 100, 91.7, 95.7, and 100%, respectively. CE was able to distinguish malignant from benign tumors with an AUC of 0.953 (95% CI: 0.870–0.990). Multivariate logistic regression analysis revealed that tumor area, base, and A1/A2 were associated with the risk of malignant tumor (OR = 1.003, 95% CI: 1.00003–1.005; OR = 22.64, 95% CI: 1.30–395.21; OR = 165.39, 95% CI: 4.68–5,850.94, respectively). When using A1/A2 > 1.28 as the only diagnostic criterion to identify the malignant tumor, AUC, sensitivity, specificity, PPV, and NPV were 0.886 (95% CI: 0.784–0.951), 80.6, 96.7, 96.7, and 80.7%, respectively.

**Conclusion:**

CE has the potential to accurately differentiate cardiac masses by combining qualitative and quantitative analyses. However, more studies with a large sample size should be conducted to further confirm these findings.

**Clinical trial registration:**

http://www.chictr.org.cn/, identifier: ChiCTR1900026809.

## Introduction

Cardiac masses have captured researchers' attention since the beginning of the field of echocardiography. Cardiac masses can be classified into non-neoplastic masses (thrombi, vegetations, calcifications, or other rare conditions), benign tumors, or malignant tumors (1, 2). Non-neoplastic masses account for 75% of all cases (3). Cardiac tumors are among the least prevalent of all tumors. The estimated prevalence of primary cardiac tumors is 0.001–0.03% (4), whereas metastatic cardiac tumors have been reported to occur 10–1,000 times as often (2.3–18.3%) (5). Primary cardiac tumors are divided into benign and malignant based on their histological characteristics (6). A previous study has demonstrated that the proportions of cardiac tumors in the left atrium, right atrium, left ventricle, right ventricle, and other sites were 34, 26, 6, 7, and 27%, respectively (7).

Cardiac masses may occur in any cardiac chamber adjacent to large blood vessels or pericardium. The treatments for cardiac tumors include surgical removal and chemoradiotherapy. The choice of treatment depends on the histopathological type, the extent of cancer invasion, and patient risk stratification (8). Early detection and accurate differentiation of cardiac masses might prolong survival and improve quality of life in affected patients. Several imaging modalities, including transthoracic echocardiography (TTE), transesophageal echocardiography (TEE), cardiac magnetic resonance (CMR), and positron emission tomography, have a crucial role in the assessment of cardiac masses (2). Given the diversity of cardiac masses, there are no guidelines or consensus regarding the best diagnostic approach. A recently published paper has comprehensively summarized the utility of these imaging modalities (9), stating that TTE is usually the first choice for cardiac mass examination. Once a cardiac mass is suspected based on TTE results, patients may be scanned using CMR for further evaluation due to the high resolution of cardiac mass boundary it provides. Positron emission tomography is helpful for staging malignancies and optimizing biopsy location.

TTE can help to determine the presence, size, shape, echogenicity, mobility, attachment point, and hemodynamic effects of the cardiac masses (10). The sensitivity of TTE to diagnose cardiac masses is 93% (11). However, it is not sufficient for some patients when image quality is suboptimal and echo are complex. With an accuracy of less than 70%, it is very challenging to differentiate between benign and malignant tumors using TTE (12). Contrast echocardiography (CE) is a rapidly developing technology in recent years. The published guidelines for CE state that it can improve the image quality and help to distinguish between benign and malignant lesions (class of recommendation: benefits are greater than risks, and the procedure can be useful if performed) (13). However, most studies on CE diagnosis of cardiac masses are case reports (14–16), while the rest are retrospective (12) or small sample-sized prospective studies (17). The evidence for differential diagnosis of cardiac masses using CE is insufficient. Therefore, the present study aimed to evaluate the diagnostic accuracy of CE in patients with suspected cardiac masses.

## Materials and methods

A group of six tertiary hospitals in North China, including the second largest hospital in China, conducted this prospective study. All the data collected were sent to Shengjing Hospital of China Medical University, as in our previous multicenter study (18). The study followed the STARD guidelines.

### Study participants

Consecutive patients with suspected cardiac masses based on TTE performed between November 2019 and December 2020 were eligible for inclusion. All patients were adults. Exclusion criteria included allergies to albumin, blood products, and ultrasound enhancing agents; severe heart failure (New York Heart Association Class IV) and severe arrhythmia patients; respiratory failure; severe liver or kidney dysfunction; and mental illness or epilepsy (19).

### Echocardiographic image acquisition

Echocardiographic examinations were performed with the patient in the left lateral position by a radiologist with more than 10 years of TTE experience using a Philips iE33 ultrasound system (Philips Medical Systems, Bothell, WA, USA) and a TTE probe (S5–1, 1–5 MHz) at each center. All images and measurements were obtained according to the echocardiography guideline (20). Subsequently, all patients were examined using CE according to the latest published guidelines (13, 21).

### Contrast echocardiography protocol

This protocol was written according to the recently published guideline for CE (13). Commercial ultrasound enhancing agents were used in the CE process (SonoVue; Bracco, Plan-Les-Ouates, Switzerland). First, the left ventricular opacification (LVO) mode was initiated with a low mechanical index of 0.2 and 30-Hz frame rates. Then, 0.8 mL of prepared ultrasound enhancing agents were quickly injected *via* the peripheral vein, with a slow (10–20 s) 3–5 mL saline flush. This was repeated as needed for optimal delineation of the left ventricular cavity and cardiac masses. The above-mentioned morphological and hemodynamic features of cardiac lesions were observed and saved digitally in this mode. Second, the myocardial contrast echocardiography (MCE) mode was turned on with a very low mechanical index of 0.08 and 30-Hz frame rates. After filling in the left ventricle and the myocardium, the ultrasound enhancing agents were continuously infused with a dedicated Vueject® syringe pump (Bracco, Milano, Italy) at a rate of 1 mL/min. The intermittent-flash (high mechanical index of 1.0) technique was used to destroy the microbubbles. Notably, the high mechanical index ultrasound impulse was transmitted between 5–10 frames to destroy the microbubbles. Perfusion was confirmed post contrast replenishment after the impulse to prevent a false positive reading due to saturation artifact. Finally, imaging results for the enhancement of the masses and adjacent normal myocardium before and after the flash were stored.

### Echocardiographic image analysis

**Figure 1:**
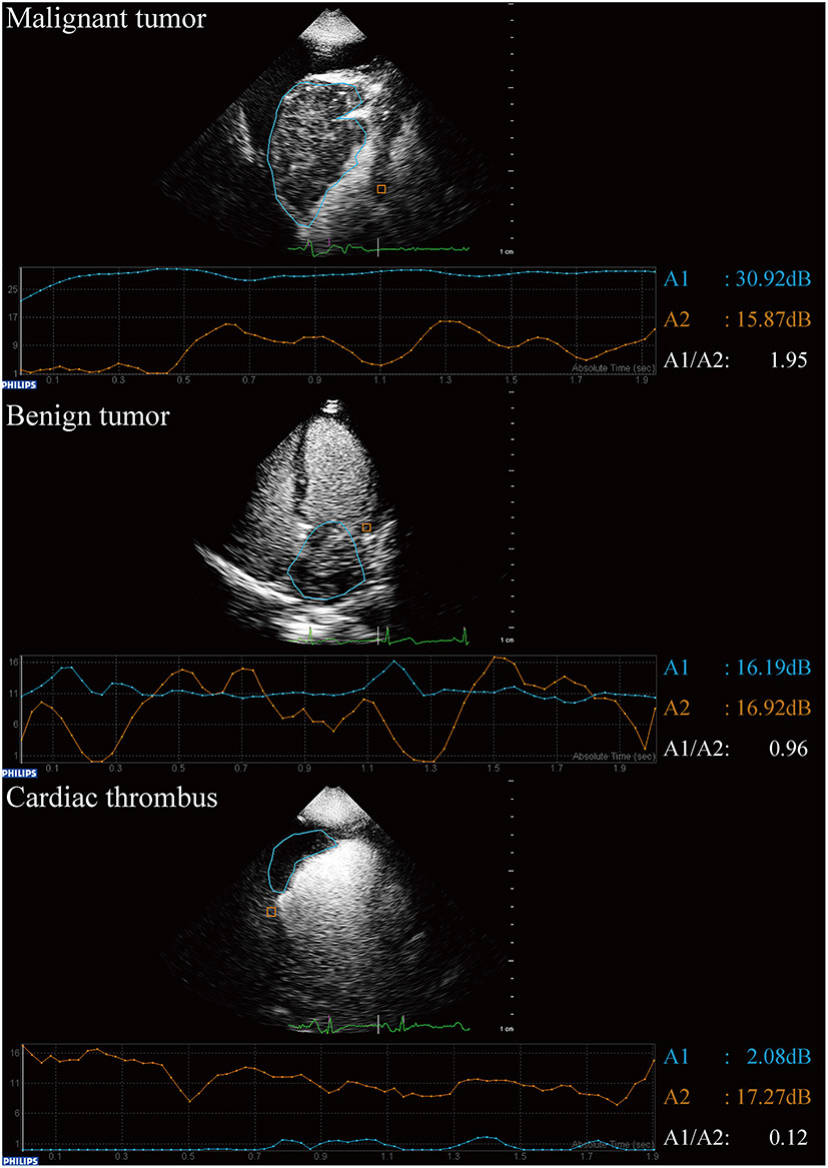
Qualitative analysis of contrast echocardiography in patients with suspected cardiac masses using QLAB software.

The qualitative analysis included echogenicity (uniform/non-uniform), boundary (well-demarcated/not well-demarcated), morphology of the base under CE (narrow with peduncle/narrow with notch/broad) (22), mass perfusion (no perfusion/mild perfusion/intense perfusion) (23), motility (absent/present) (24), and pericardial effusion (absent/present). Quantitative analysis was performed using QLAB software (version 13.0; Philips Medical Systems, Andover, MA, USA). The area of the masses was measured when the long maximum diameter was apparent. The region of interest was depicted along the boundary of each lesion and within the adjacent myocardium section (23). The peak intensity of the masses and of the adjacent myocardium were measured as A1 and A2, respectively (Figure 1) (25). The ratio of A1 to A2 was then calculated (26). A1/A2 > 1 was considered to indicate a high possibility of malignant tumor, while a mass with a ratio between 0 and 1 was considered to be a benign tumor or thrombus (26, 27). The mass with A1 close to zero was considered a thrombus.

All analyses were performed independently by two investigators (Y.L. and X.W.) with more than 6 years of experience with CE and more than 10 years of experience with TTE. To improve the specificity of CE differentiation of malignant tumor from benign tumor based on the pilot study, the final diagnosis was made based on the combination of qualitative and quantitative results (26). Disagreements were discussed and resolved by involving a senior CE expert (W.R.) for adjudication.

### Follow-up and validation

All patients were followed up to determine all-cause mortality by checking their medical records, performing telephone interviews, and *via* outpatient exams every 6 months until March 1, 2022. (I) Pseudomass was defined as a variant or prominent normal structure, including Eustachian valve or Chiari network, Crista terminalis, and Coumadin ridge (28). Diagnosis was confirmed by CMR. No morphological changes were identified by follow-up imaging. (II) Thrombus was defined as a distinct mass of echoes that can be seen throughout systole and diastole (29). Either of the following two criteria had to be met: i) a significantly diminishing size or full resolution after anticoagulation therapy and confirmation of thrombus upon follow-up TEE or computed tomography (CT); or ii) pathological confirmation (24). (III) All tumors had to be confirmed by surgery or biopsy. Tumors were classified as benign or malignant based on histology (pathologic analysis) results in accordance with the 2015 World Health Organization classification of tumors of the heart and pericardium (6).

### Measurement variability

To determine the intra-observer variability for all qualitative and quantitative indexes, measurements of 50 randomly selected cases were reassessed 2 weeks later by an investigator (Y.X.) who was blinded to the previous measurement results. To determine the inter-observer variability, measurements were repeated by a second observer (Y.F.) who was blinded to the results obtained by the first investigator. The two investigators were equally experienced.

### Sample size calculation

The appropriate sample size was estimated with a 5% level of type I error and a minimal statistical power of 80% using PASS software (PASS 21.0.3. NCSS, LLC, Kaysville, UT, USA). The average sensitivity was 92.9% and the average specificity was 78.3% when using CE to differentiate malignant tumors from benign tumors based on Xia et al. (12) and our previous study (26) findings. When the prevalence/ratio was 35.0% (number of patients with malignant tumor/number of total cardiac tumor patients), the test required a minimum sample size of 46 (16 participants with malignant tumors and 30 participants with benign tumors).

The sensitivity was greater than 88.9% and specificity was greater than 80% when using CE to differentiate cardiac tumors from non-neoplastic cardiac masses in our previous study (26). When the prevalence/ratio was 57.1% (number of patients with thrombus/total number of patients with cardiac mass) (26), a sample size of 65 participants (37 participants with tumors and 28 participants with non-neoplastic cardiac masses) were sufficient to differentiate cardiac tumors from non-neoplastic cardiac masses. Therefore, 81 (16 malignant tumors, 30 benign tumors, and 35 non-neoplastic cardiac masses) was the minimum sample size for this study.

### Statistical analysis

Continuous parameters were expressed as the mean ± standard deviation, and differences between groups were analyzed using independent-samples *t*-tests. Non-normally distributed parameters were expressed as the median (interquartile range, IQR), and differences between groups were analyzed using the Mann-Whitney *U* test. Comparison of categorical parameters between groups was analyzed using Pearson's chi-squared test or Fisher's exact test. Univariate logistic regression analysis was performed to evaluate the association between different echocardiographic parameters of cardiac tumors. Additionally, multivariate analysis was performed with the identified significant variables (*P* < 0.05). Odds ratio (OR) and 95% confidence intervals (CIs) were also calculated. Receiver operating characteristic (ROC) analysis was conducted to assess the differentiating capacity of variables for cardiac masses. Youden's J statistic was used to determine the optimal cutoff value. Finally, the area under the receiver operating characteristic curve (AUC), accuracy, sensitivity, specificity, positive predictive value (PPV), and negative predictive value (NPV) were calculated. Interclass correlation coefficient was used for continuous variables, and weighted kappa (**κ_w_**) was used for categorical measurements. A *P* value of <0.05 was used to define statistical significance. Statistical analyses were performed using Stata (version 16.0; StataCorp, College Station, TX, USA).

## Results

### Population characteristics

**Figure 2:**
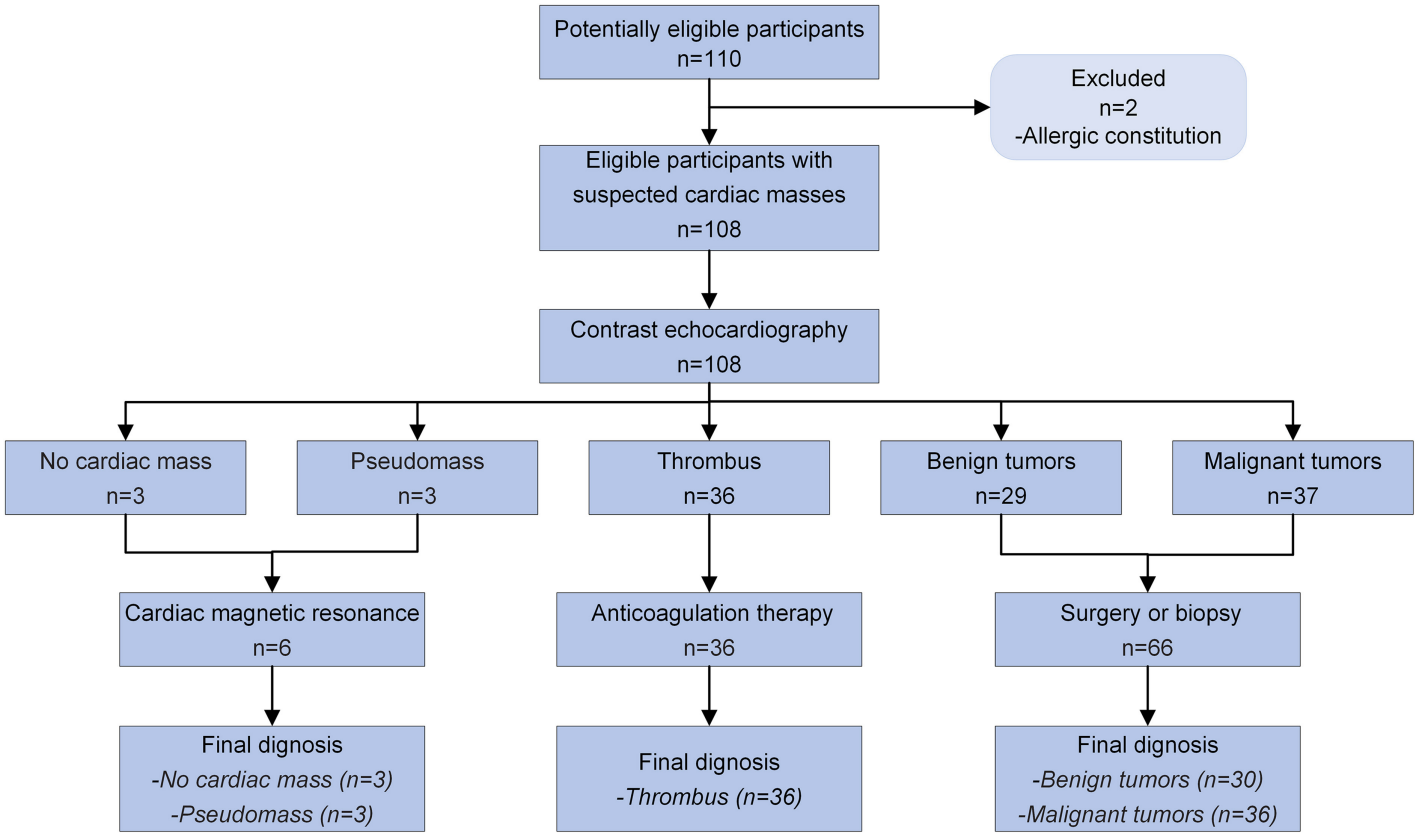
Diagnostic flow diagram for patients with suspected cardiac masses using contrast echocardiography according to STARD 2015.

A total of 46,111 TTEs were performed at six departments between November 1, 2019 and December 31, 2020. During this period, 110 (0.24%) examinations were carried out in patients with suspected cardiac masses. Two patients with allergic constitution refused CE (Figure 2). As a result, 108 patients with a median age of 61.5 years (IQR: 52.0–67.5 years) were enrolled in the study, of which 68 (63.0%) were men. The baseline demographic and clinical characteristics of all patients are summarized in Table 1. In these 108 patients: three patients did not have any cardiac masses, three patients had a cardiac pseudomass, 36 patients had a cardiac thrombus, 30 patients had a benign tumor, and 36 patients had a malignant tumor. These results revealed that the history of previous cardiovascular disease and malignancy were significantly different among the four groups.

**Table 1:** Characteristics of the population.

	**No cardiac mass** **(*n* =3) and pseudomass (*n* =3)**	**Thrombus (*n =* 36)**	**Benign tumor (*n =* 30)**	**Malignant tumor (*n =* 36)**	***P* value**
Age, mean (SD), years	58.7 (10.7)	57.1 (14.7)	55.1 (13.3)	65.1 (9.8)	0.117
Sex (Male/Female)					0.810
Male	4	21	21	22	
Female	2	15	9	14	
BMI, mean (SD), kg/m^2^	24.5 (0.8)	24.6 (2.1)	23.6 (2.1)	24.0 (2.0)	0.153
Symptom					0.069
Asymptomatic	4	8	5	2	
Dyspnea	0	13	11	21	
Chest pain	0	7	6	5	
Palpitations	1	4	2	3	
Others	1	4	6	5	
History of cardiovascular disease	6	33	18	19	<0.001
History of malignant disease	0	2	3	33	<0.001
Localization					0.084
Left ventricle	3	4	0	1	
Left atrium	0	5	8	1	
Right ventricle	0	8	5	9	
Right atrium	1	12	13	19	
Others^*^	2	7	4	6	

SD, standard deviation;

* details are shown in the Supplementary Table S3.

Two cases of cardiac pseudomass were hypertrophy of the interatrial septum, and one case of cardiac pseudomass was hypertrophy of papillary muscle. All patients with a cardiac thrombus received anticoagulation therapy, and none underwent a pathological analysis. Thrombi were all solitary. A total of 75% (27/36) of the thrombi were dissolved, and in 25% of cases (9/36) the thrombus volume was significantly reduced. Benign tumors were confirmed by surgery (28/30) and biopsy (2/30). Malignant tumors were confirmed by surgery (all three were primary malignant tumors) and biopsy (27/30). The diagnoses made by two investigators (YL and XW) were consistent in 104 cases. Four controversial cases were discussed and diagnosed with the help of a senior CE expert (W.R.): one case in the thrombus group with A1/A2 of 2.12 (correct diagnosis before treatment); two cases in the benign group with A1/A2 of 1.21 and 1.16 (erroneous diagnoses before treatment); and one case in the malignant group with A1/A2 of 0.54 (erroneous diagnosis before treatment). More details on the location and histopathology of cardiac tumors are shown in Supplementary Tables S1, S2. No adverse drug reactions were observed in any of the 108 patients.

### Comparison and differentiation of cardiac tumors from thrombi

Compared to the thrombus group, a larger area, higher rate of non-uniform echogenicity, wider base, higher perfusion intensity, and higher A1/A2 were identified in the tumor group (*P* < 0.05; Table 2). Multivariate regression analysis revealed that the base and enhancement A1/A2 were associated with the presence of cardiac tumor compared to the thrombus (OR = 7.53, 95% CI: 1.10–51.56; OR = 20.09, 95% CI: 4.17–96.72, respectively; Table 2). The AUC for A1/A2 was 0.958 (95% CI: 0.899–0.988) when the cutoff value for A1/A2 was set to 0.295. The accuracy, sensitivity, specificity, PPV, and NPV are shown in Table 3.

**Table 2:** Comparison of echocardiographic parameters between thrombus and tumor.

	**Thrombus** **(*n =* 36)**	**Tumor** **(*n =* 66)**	***P* value**	**Univariate regression**		**Multivariate regression** ^*^	
				**OR (95%CI)**	***P* value**	**OR (95%CI)**	***P* value**
Area, mean (SD), mm^2^	966.5 (378.4)	1,484.5 (783.4)	<0.001	1.001 (1.000–1.002)	0.001	1.00 (0.99–1.01)	0.188
Echogenicity			0.008	3.08 (1.31–7.21)	0.010	2.31 (0.51–10.43)	0.278
Uniform	24	26					
Non-uniform	12	40					
Boundary			0.143	1.95 (0.79–4.80)	0.147		
Well-demarcated	27	40					
Not well-demarcated	9	26					
Base			<0.001	21.69 (4.81–97.86)	<0.001	7.53 (1.10–51.56)	0.040
Narrow with peduncle	0	17					
Narrow with notch	34	12					
Broad	2	37					
Mass perfusion			<0.001	0.24 (0.06–0.93)	0.040	1.44 (0.18–11.38)	0.731
No perfusion	23	0					
Mild perfusion	10	29					
Intense Perfusion	3	37					
Motility			0.097	2.33 (0.84–6.46)	0.103		
Absent	30	45					
Present	6	21					
Pericardial effusion			0.275	1.71 (0.69–4.24)	0.244		
Absent	27	42					
Present	9	24					
Enhancement A1/A2, median (IQR)	0.05 (0.04–0.17)	1.16 (0.71–1.88)	<0.001	38.82 (8.33–180.93)	<0.001	20.09 (4.17–96.72)	<0.001

CI, confidence interval; IQR, interquartile range; OR, odds ratio; SD, standard deviation.

* variables entered into the multivariate regression included area, echogenicity, base, mass perfusion, and enhancement A1/A2.

**Table 3:** Comparison of diagnostic performance in differentiating thrombus from cardiac tumor.

	**Sensitivity**	**Specificity**	**AUC**	**Accuracy**	**PPV**	**NPV**
Combined qualitative and quantitative analysis	100% (94.6%−100%)	100% (90.3%−100%)	1.000 (0.964–1.000)	100% (96.5%−100%)	100%	100%
Using A1/A2 alone (Cutoff value = 0.295)	100% (94.6%−100%)	91.7% (77.5%−98.3%)	0.958 (0.899–0.988)	97.1% (91.6%−99.4%)	95.7% (88.2%−98.5%)	100%

AUC, the area under the receiver operating characteristic curve; NPV, negative predictive value; PPV, positive predictive value.

### Comparison and differentiation of malignant tumors from benign tumors

Compared to the benign group, a larger area, higher rate of non-uniform echogenicity, not well-demarcated boundary, wider base, presence of motility, and higher A1/A2 were identified in the tumor group (*P* < 0.05; Table 4). Multivariate regression analysis revealed that the area, base, and A1/A2 were associated with the presence of malignant tumor compared to the benign tumor (OR = 1.003, 95% CI: 1.00003–1.005; OR = 22.64, 95% CI: 1.30–395.21; OR = 165.39, 95% CI: 4.68–5,850.94, respectively; Table 4). When the cutoff value for A1/A2 was set to 1.28, the AUC for A1/A2 was 0.886 (95% CI: 0.784–0.951). When the cutoff value for the tumor area was set to 1,302.2 mm^2^, the AUC for the tumor area was 0.725 (95% CI: 0.601–0.828). The accuracy, sensitivity, specificity, PPV, and NPV are shown in Table 5.

**Table 4:** Comparison of echocardiographic parameters between malignant tumor and benign tumor.

	**Benign tumor** **(*n =* 30)**	**Malignant tumor** **(*n =* 36)**	***P* value**	**Univariate regression**		**Multivariate regression** ^*^	
				**OR (95%CI)**	***P* value**	**OR (95%CI)**	***P* value**
Area, mean (SD), mm^2^	1,153.98 (721.68)	1,759.86 (732.47)	<0.001	1.001 (1.0004–1.002)	0.003	1.003 (1.00003–1.005)	0.023
Echogenicity			0.034	2.97 (1.07–8.26)	0.037	2.30 (1.00003–1.005)	0.487
Uniform	16	10					
Non-uniform	14	26					
Boundary			0.015	3.67 (1.26–10.70)	0.017	24.46 (0.94–636.52)	0.055
Well-demarcated	23	17					
Not well-demarcated	7	19					
Base			<0.001	16.43 (4.86–55.55)	<0.001	22.64 (1.30–395.21)	0.033
Narrow with peduncle	15	2					
Narrow with notch	8	4					
Broad	7	30					
Mass perfusion			0.057	2.62 (0.96–7.12)	0.060		
Mild perfusion	17	12					
Intense Perfusion	13	24					
Motility			0.018	0.28 (0.09–0.82)	0.021	2.05 (0.15–28.56)	0.592
Absent	16	29					
Present	14	7					
Pericardial effusion			0.071	2.94 (0.94–8.57)	0.077		
Absent	23	19					
Present	7	17					
Enhancement A1/A2, median (IQR)	0.73 (0.25)	1.76 (0.61)	<0.001	84.07 (10.18–694.37)	<0.001	165.39 (4.68–5850.94)	0.005

CI, confidence interval; IQR, interquartile range; OR, odds ratio; SD, standard deviation.

* variables entered into the multivariate regression included area, echogenicity, boundary, base, motility, and enhancement A1/A2.

**Table 5:** Comparison of diagnostic performance in differentiating malignant tumor from benign tumor.

	**Sensitivity**	**Specificity**	**AUC**	**Accuracy**	**PPV**	**NPV**
Combined qualitative and quantitative analysis	97.2% (85.5–99.9%)	93.3% (77.9–99.2%)	0.953 (0.870–0.990)	95.5% (87.3–99.1%)	94.6% (82.1–98.5%)	96.6% (80.2–99.5%)
Area alone (Cutoff value = 1,302.2)	75.0% (57.8–87.9%)	70.0% (50.6–85.3%)	0.725 (0.601–0.828)	72.7% (60.4–83.0%)	75.0% (62.7–84.2%)	70.0% (55.8–81.1%)
Using A1/A2 alone (Cutoff value = 1.00)	83.3% (67.2–93.6%)	83.3% (65.3–94.4%)	0.833 (0.721–0.914)	83.3% (72.1–91.4%)	85.7% (72.7–93.1%)	80.6% (66.4–89.8%)
Using A1/A2 alone (Cutoff value = 1.28)	80.6% (64.0–91.8%)	96.7% (82. 8–99.9%)	0.886 (0.784–0.951)	87.9% (77.5–94.6%)	96.7% (80.7–99.5%)	80.7% (68.0–89.0%)

AUC, the area under the receiver operating characteristic curve; NPV, negative predictive value; PPV, positive predictive value.

### Follow-up

**Figure 3:**
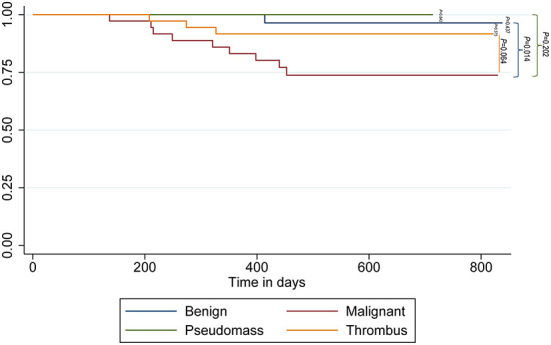
Kaplan-Meier survival curve for patients in this study.

The median follow-up duration was 570 days (IQR: 447–691 days). The 1-year survival rates for no mass/pseudomass, thrombus, benign tumor, and malignant tumor groups were 100, 88.9, 100, and 80.6%, respectively. Patients in the malignant group had a lower survival rate compared to patients in the benign group (*P* = 0.014, Figure 3).

### Reproducibility

The inter- and intra-observer reproducibility values were excellent for all qualitative and quantitative indexes (Supplementary Table S3).

## Discussion

CE is a useful tool for patients with suspected cardiac masses. In the present study, the diagnostic sensitivity and specificity of CE were high when differentiating cardiac tumors from non-neoplastic cardiac masses. At the same time, CE was superior to conventional TTE and comparable to pathologic analysis for differentiating malignant tumors from benign tumors. To the best of our knowledge, this is the first multicenter diagnostic study of CE in patients with suspected cardiac masses.

Cardiac masses are often encountered during clinical practice. They can be serious or even life-threatening. Improving the diagnostic efficiency of cardiac mass is an urgent goal of radiologists. TTE, transesophageal echocardiography, and CMR are commonly used in diagnostic procedures. Currently, CMR is the hottest topic in cardiac mass research. Several retrospective studies have shown that the CMR features demonstrate excellent accuracy for the differentiation of cardiac thrombi from tumors and can be helpful for the distinction of benign and malignant neoplasms (24, 30–34). A prospective CMR study has demonstrated that tumor size (>3.25 cm), invasion, and first-pass perfusion are useful imaging characteristics in differentiating benign from malignant tumors (35). Another prospective CMR study has revealed that invasiveness, irregular border, and late heterogeneous gadolinium enhancement are better variables for differentiating benign and malignant tumors (36). However, all of the above studies were limited to qualitative or semi-quantitative analysis. Therefore, a diagnostic imaging technique with quantitative parameters is urgently needed to share the burden of CMR and to reduce pathology specialist workload.

At present, TTE is still the first diagnostic procedure used to evaluate cardiac masses. Conventional TTE usually assesses the following characteristics of cardiac masses: site, base, mortality, and echogenicity. However, cardiac tumors, especially differentiating benign and malignant tumors, are very challenging to analyze using conventional TTE. An accurate diagnosis based on qualitative information depends more on the experience of the radiologist. To address this issue, CE has become an indispensable part of echocardiography with rapid development in the recent decade (37). The application of ultrasound enhancing agents based on conventional TTE can clearly display the endocardial boundary of the left ventricle and improve the accuracy of the left ventricular ejection fraction measurement (13). CE data can also be quantitatively analyzed. Kirkpatrick et al. have published the first study that demonstrated the diagnostic utility of A1 and A2 values using CE in cardiac masses in 2004 (38). Since then, several radiologists have shared their diagnosis experiences using CE to identify cardiac masses (12, 17, 23, 26, 27, 39–41). Five of these studies have provided the evidence for the indispensable differential diagnostic value of A1/A2 (12, 26, 27, 38, 40). Xia et al. have found a significant difference in A1/A2 between malignant and benign tumors (1.34 ± 0.43 vs. 0.65 ± 0.17, *P* <0.01) (12). Mao et al. have revealed that A1/A2 >1 had a high diagnostic accuracy in differentiation of a benign mass from a malignant metastatic tumor in a cohort study (27). Furthermore, A1/A2 >1 was a significant and independent predictor of future death in patients with cardiac masses and a history of extracardiac malignant tumors (27).

### Differentiation between cardiac tumors and thrombi

The present study found that CE had an excellent accuracy in the diagnosis of intracardiac thrombi. Setting A1/A2 with a cutoff value of 1 had a 91.7% specificity and nearly 100% sensitivity when diagnosing a thrombus. Interestingly, the A1/A2 value for most thrombi was around zero. However, three cases had a much higher A1/A2 (1.91, 2.12, and 1.85, respectively), likely because these three cases were fresh thrombi. The loose texture of a fresh thrombus and the ability of the ultrasound-enhancing agents to enter from the periphery at the beginning of CE result in a higher A1/A2, which is consistent with a previous study (19). Conversely, the texture of an old thrombus is dense, and the microbubbles of the ultrasound-enhancing agents cannot enter, resulting in A1/A2 values close to zero. Differentiation of a fresh thrombus from an old thrombus has important clinical value: a fresh thrombus is easier to remove than an old one (42). A fresh thrombus is also less fixed to the left ventricular wall and more fragile because of its collagen-poor organization (43). The risk of fresh thrombus shedding should be evaluated with great care.

Another perfusion phenomenon was demonstrated by Uenishi et al. (23). They found that ultrasound-enhancing agents often do not enter the interior of the thrombus (81.8%, 27/33) or only stay at its periphery (12.1%, 4/33). The ultrasound enhancing agents usually perfuse the periphery of the cardiac tumor (44.7%, 21/47) or even the entire tumor (48.9%, 23/47). The perfusion patterns identified in the present study and the findings by Uenishi et al. require more samples to confirm in the future (23).

### Differentiation between cardiac tumors and thrombi

The present study combined CE quantitative parameters with qualitative echocardiographic assessment to improve the diagnostic accuracy of cardiac tumors compared to our previous study (26). The resulting accuracy was comparable to that of CMR (accuracy = 98.4%) (34).

CE can improve the image quality and assess the blood supply inside the tumor. Benign tumors often have sparse blood supply, while malignant tumors have rich blood supply (44, 45). Previous studies have usually used 1.0 as the cutoff value for A1/A2 to differentiate malignant tumors from benign tumors (26, 38, 40). However, benign tumors may have an A1/A2 which is close to or slightly more than 1 [1.32 in a hemangioma (38), 1.08 in a rhabdomyoma, 0.84 in a fibroma, 0.92 in a hemangioma (40), and 1.06–1.15 in myxomas (26)]. Some malignant tumors containing necrotic tissue result in an A1/A2 of <1 [3.6%, 2/55 in Mao et al. (27)]. The present results revealed that 1.28 is better than 1 as the cutoff value for A1/A2 to differentiate malignant tumors from benign tumors. For less experienced radiologists, only using A1/A2 with a cutoff value of 1.28 would achieve a good diagnostic result. The tumor area/size is also helpful during differentiation, which is consistent with a previous study (35).

**Figure 4:**
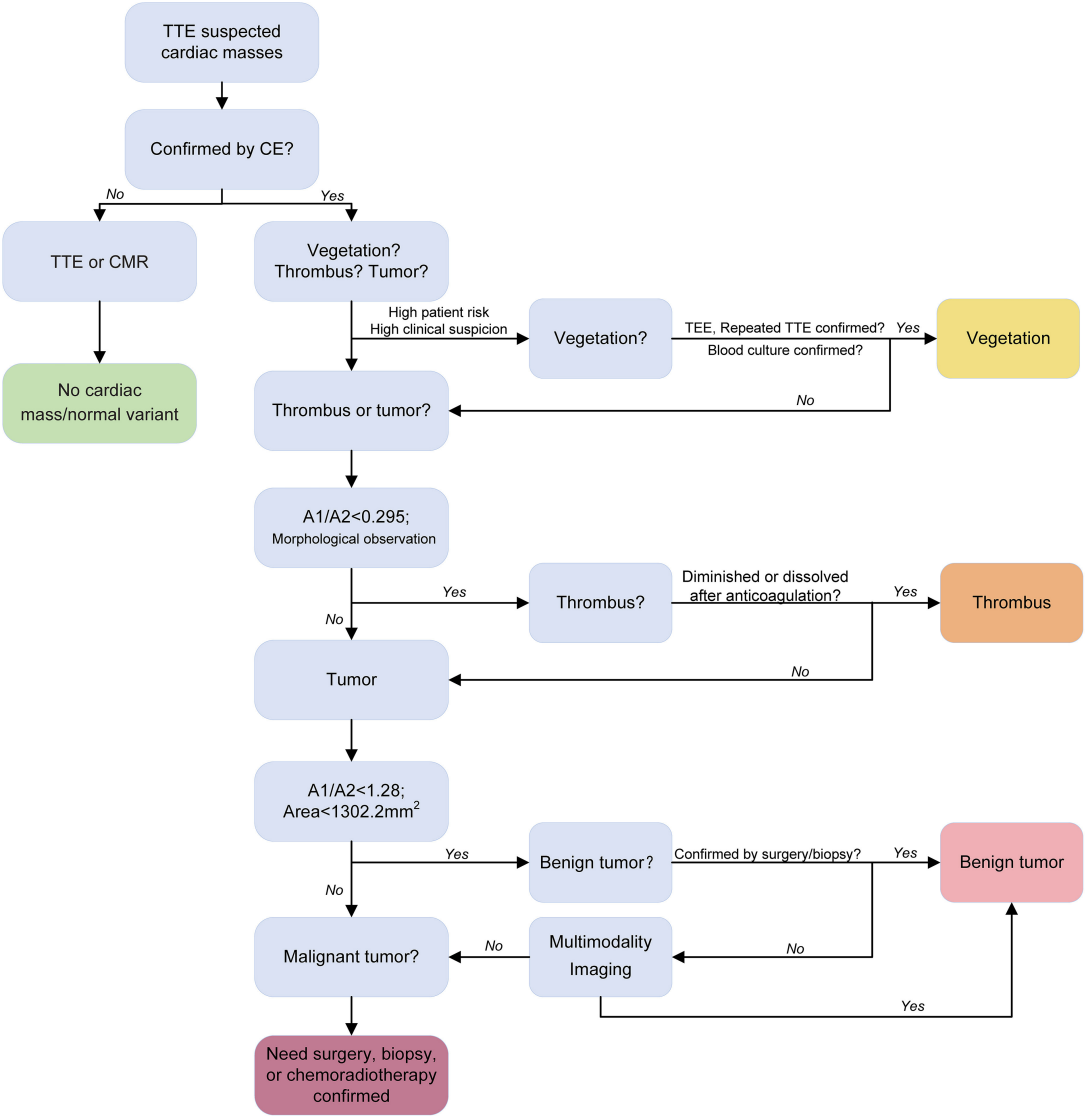
Proposed diagnostic flow of cardiac tumors using contrast echocardiography. Transthoracic echocardiography and contrast echocardiography identified the cardiac mass (red arrow).

The strengths of the present study include the novelty of the diagnostic approach to differentiate cardiac masses, prospective study design, and relatively large sample size. Simple, quick, highly repeatable quantitative parameter (A1/A2) is of great help for clinical diagnosis, especially for radiologists who do not have much experience in diagnosing cardiac masses using TTE. The proposed diagnostic flow for cardiac tumors using CE is shown in Figure 4.

### Other modalities

TEE can also be useful in the diagnosis of cardiac masses. Several previous studies have shown that the use of ultrasound-enhancing agents can increase the diagnostic accuracy of cardiac thrombi in a TEE exam for atrial fibrillation patients (46–48). Xia et al. have found that the combination of TEE and CE was feasible for the detection of suspected cardiac masses with an accuracy of 97.8–100%, especially in diagnosing and differentiating between benign and malignant lesions (12).

Cardiac CT may offer an alternative to CMR, especially when other imaging modalities are non-diagnostic or contraindicated (9, 49). Compared to other cardiac imaging modalities, cardiac CT is optimal for the evaluation of calcified masses (9, 49). Disadvantages of cardiac CT include radiation exposure, low risk of contrast-induced nephropathy, and limited soft tissue and temporal resolutions compared to magnetic resonance imaging (9). Several studies have revealed that cardiac CT can differentiate between cardiac tumors and thrombi (50–53). A prospective study with a large sample is needed to confirm this finding.

### Limitations

The present study had several limitations. First, the study only included 36 patients with a thrombus and 66 patients with a cardiac tumor. The incidence of primary malignant cardiac tumor is extremely low. Only three cases were included in the study. Therefore, the limited spectrum of the cardiac tumor represents a limitation. Second, the participating hospitals in this study were tertiary. Intracardiac thrombus with a well-demarcated boundary, broad base, and low echocardiographic suspicion is usually treated at secondary hospitals instead of transferring to our hospitals. Therefore, most thrombi in the present study were atypical and without a broad base. Third, due to the low number of pseudomass cases in the study, more cases need to be included in future analyses. Finally, the recruitment period for patients was short. Long-term follow-up may be needed to determine whether A1/A2 can predict the prognosis for patients with cardiac tumors. Fourth, the study analysis did not explore the diagnostic performance of CE performed by less experienced radiologists (54). The experience with CE may be an underlying confounder in this study.

## Conclusion

In summary, CE has the potential to accurately differentiate cardiac masses by combining qualitative and quantitative analyses. However, more studies with a large sample size should be conducted to further confirm the present study findings.

## Data availability statement

The original contributions presented in the study are included in the article/Supplementary material, further inquiries can be directed to the corresponding author/s.

## Ethics statement

The studies involving human participants were reviewed and approved by the Ethics Committee of Shengjing Hospital of China Medical University. Written informed consent to participate in this study was provided by the participants' legal guardian/next of kin. Written informed consent was obtained from the individual(s), and minor(s)' legal guardian/next of kin, for the publication of any potentially identifiable images or data included in this article.

## Author contributions

All authors listed have made a substantial, direct, and intellectual contribution to the work and approved it for publication.

## Funding

This study has received funding by the New Medical Technology Project of Shengjing Hospital (2017–029).

## Conflict of interest

The authors declare that the research was conducted in the absence of any commercial or financial relationships that could be construed as a potential conflict of interest.

## Publisher's note

All claims expressed in this article are solely those of the authors and do not necessarily represent those of their affiliated organizations, or those of the publisher, the editors and the reviewers. Any product that may be evaluated in this article, or claim that may be made by its manufacturer, is not guaranteed or endorsed by the publisher.
