# Supplementary material for: The diagnostic accuracy of contrast echocardiography in patients with suspected cardiac masses: A preliminary multicenter, cross-sectional study
Source: Front Cardiovasc Med. 2022 Sep 16;9:1011560. doi: 10.3389/fcvm.2022.1011560 (PMC9523017; doi:10.3389/fcvm.2022.1011560)
Supplement: Supplementary file 1 [file Data_Sheet_1.PDF]

The diagnostic accuracy of contrast echocardiography in patients with suspected cardiac masses: a preliminary, multicenter, cross-sectional study

Content

Table S1 The cardiac tumor distribution 2

Table S2 The summary of special site of cardiac masses 2

Table S3 Agreement for contrast echocardiography assessment 2

STARD 2015 3

Table S1 The cardiac tumor distribution

Benign tumors (n = 30)	Numbers
Myxoma	21
Hemangioma	3
Intravenous leiomyomatosis	2
Lipoma	2
Fibroma	1
Fibroelastoma	1
Malignant tumors (n = 36)	
Primary mesothelioma	1
Primary angiosarcoma	2
Metastasis of lung cancer unspecified	14
Metastasis of lung cancer, non-small cell	4
Metastasis of liver cancer	4
Metastasis of oophoroma	3
Metastasis of breast cancer	2
Metastasis of hepatocellular carcinoma	2
Metastasis of pancreatic cancer	2
Metastasis of renal cell carcinoma	2

Table S2 The summary of special site of cardiac masses

	Pseudomass	Thrombus	Benign tumor	Malignant tumor
Left ventricle apex	0	2	0	0
Ventricular septum	2	0	0	0
Left atrium appendage	0	7	0	0
Aortic valve	0	0	1	0
Mitral valve	0	0	1	0
Tricuspid valve	0	0	1	0
Pulmonary artery	0	0	1	6

Table S3 Agreement for contrast echocardiography assessment

	Intra-observer	Inter-observer
Area	ICC: 0.972 (0.951–0.984)	ICC: 0.928 (0.877–0.959)
Echogenicity	K_w: 0.718 (0.526–0.910)	K_w:0.760 (0.579–0.940)
Boundary	K_w: 0.766 (0.573–0.960)	K_w: 0.757 (0.557–0.958)
Base	K_w: 0.822 (0.694–0.950)	K_w: 0.826 (0.696–0.955)
Mass perfusion	K_w: 0.769 (0.607–0.932)	K_w: 0.754 (0.584–0.923)
Motility	K_w: 0.860 (0.709–0.999)	K_w: 0.715 (0.503–0.927)
Pericardial effusion	K_w: 0.766 (0.573–0.960)	K_w: 0.757 (0.557–0.958)
Enhancement A1/A2	ICC: 0.945 (0.905–0.968)	ICC: 0.946 (0.906–0.969)

ICC: Interclass correlation coefficient; K_w: Weighted kappa.

STARD 2015

Section & Topic	No	Item	Reported on page #
**TITLE OR ABSTRACT**			
	**1**	Identification as a study of diagnostic accuracy using at least one measure of accuracy (such as sensitivity, specificity, predictive values, or AUC)	**Page 1**
**ABSTRACT**			
	**2**	Structured summary of study design, methods, results, and conclusions (for specific guidance, see STARD for Abstracts)	**Page 1-2**
**INTRODUCTION**			
	**3**	Scientific and clinical background, including the intended use and clinical role of the index test	**Page 2**
	**4**	Study objectives and hypotheses	**Page 2**
**METHODS**			
Study design	**5**	Whether data collection was planned before the index test and reference standard were performed (prospective study) or after (retrospective study)	**Page 2-3**
Participants	**6**	Eligibility criteria	**Page 3**
	**7**	On what basis potentially eligible participants were identified (such as symptoms, results from previous tests, inclusion in registry)	**Page 3**
	**8**	Where and when potentially eligible participants were identified (setting, location and dates)	**Page 3**
	**9**	Whether participants formed a consecutive, random or convenience series	**Page 3**
Test methods	**10a**	Index test, in sufficient detail to allow replication	**Page 3-5**
	**10b**	Reference standard, in sufficient detail to allow replication	**Page 3-5**
	**11**	Rationale for choosing the reference standard (if alternatives exist)	**Page 3-5**
	**12a**	Definition of and rationale for test positivity cut-offs or result categories of the index test, distinguishing pre-specified from exploratory	**Page 3-5**
	**12b**	Definition of and rationale for test positivity cut-offs or result categories of the reference standard, distinguishing pre-specified from exploratory	**Page 3-5**
	**13a**	Whether clinical information and reference standard results were available to the performers/readers of the index test	**Page 3-5**
	**13b**	Whether clinical information and index test results were available to the assessors of the reference standard	/
Analysis	**14**	Methods for estimating or comparing measures of diagnostic accuracy	**Page 3-5**
	**15**	How indeterminate index test or reference standard results were handled	**Page 3-5**
	**16**	How missing data on the index test and reference standard were handled	**Figure 1**
	**17**	Any analyses of variability in diagnostic accuracy, distinguishing pre-specified from exploratory	**Page 3-5**
	**18**	Intended sample size and how it was determined	**Page 4**
**RESULTS**			
Participants	**19**	Flow of participants, using a diagram	**Figure 1**
	**20**	Baseline demographic and clinical characteristics of participants	**Page 5-6**
	**21a**	Distribution of severity of disease in those with the target condition	**Page 5-6**
	**21b**	Distribution of alternative diagnoses in those without the target condition	**Page 5-6**
	**22**	Time interval and any clinical interventions between index test and reference standard	**Page 5-6**
Test results	**23**	Cross tabulation of the index test results (or their distribution) by the results of the reference standard	**Figure 1**
	**24**	Estimates of diagnostic accuracy and their precision (such as 95% confidence intervals)	**Page 5-6**
	**25**	Any adverse events from performing the index test or the reference standard	**Page 5-6**
**DISCUSSION**			
	**26**	Study limitations, including sources of potential bias, statistical uncertainty, and generalisability	**Page 6-8**
	**27**	Implications for practice, including the intended use and clinical role of the index test	**Page 6-8**
**OTHER INFORMATION**			
	**28**	Registration number and name of registry	**Page 2**
	**29**	Where the full study protocol can be accessed	**Page 2**
	**30**	Sources of funding and other support; role of funders	**Page 8-9**
